# From molecular precision to the whole patient: New developments and therapies in neuro-oncology

**DOI:** 10.1016/j.neurot.2026.e01102

**Published:** 2026-10-15

**Authors:** Mary Jane Lim-Fat, Patrick Y. Wen

**Affiliations:** a Division of Neurology, Department of Medicine, Sunnybrook Health Sciences Centre, University of Toronto, Toronto, ON, Canada; b Center for Tumors of the Nervous System, Massachusetts General Brigham Cancer Institute, Harvard Medical School, Boston, MA, USA

The past decade has witnessed a profound transformation in neuro-oncology. The integration of molecular features into the World Health Organization classification of central nervous system (CNS) tumors, the approval of the first targeted therapy for a diffuse glioma, the clinical translation of cellular immunotherapies, and new technologies to circumvent the blood–brain barrier have all occurred in rapid succession. Nevertheless, outcomes for many patients, most notably those with IDH-wildtype glioblastoma, remain largely unchanged. This special issue of *Neurotherapeutics*, entitled New Developments and Therapies in Neuro-Oncology, comprises twelve contributions that appraise the current state of the field and identify where further therapeutic advances are most likely to emerge.

Accurate diagnosis remains the foundation of rational therapy. Eaton and colleagues review recent updates to diagnostic and prognostic molecular biomarkers across CNS tumors [1]. These updates include cIMPACT-NOW refinements to the grading of IDH-mutant astrocytoma, the caution warranted when TERT promoter mutation, EGFR amplification or chromosome +7/−10 is used in isolation to diagnose glioblastoma in younger patients, and emerging platforms that generate genomic and methylation data within a single assay.

Diagnostic precision confers clinical benefit only when accompanied by rigorous therapeutic evaluation. Wisdom and Rahman contend that trial design has itself slowed progress: only 8–11% of patients with newly diagnosed glioblastoma enroll in trials, and single-arm phase 2 studies benchmarked against historical controls have repeatedly failed to predict phase 3 outcomes [2]. They examine master protocols, Bayesian adaptive designs, external control arms and window-of-opportunity studies, while acknowledging that platform trials have not yet identified a practice-changing therapy for glioma.

Maximal safe resection remains the initial intervention for most diffuse gliomas. Kueckelhaus and colleagues summarize the RANO resect classification, which shifts emphasis from relative extent of resection to absolute residual tumor volume. They review the evidence for supramaximal resection, and how functional mapping, fluorescence guidance, stimulated Raman histology and intraoperative molecular assessment are redefining both the oncological and functional objectives of surgery [3].

In IDH-mutant glioma, the approval of vorasidenib on the basis of the phase 3 INDIGO trial represents a substantive advance. Nakhate, Youssef and Wen contextualize mutant IDH inhibition alongside radiotherapy and chemotherapy, delineate questions that remain unresolved in grade 3 and contrast-enhancing disease, and review strategies directed at DNA damage repair, metabolic dependencies and antitumor immunity [4].

Glioblastoma, in contrast, continues to be characterized by a paucity of effective therapies. Blaquier and colleagues review targeted approaches for BRAF- and FGFR-altered tumors, oncolytic and retroviral vectors, microenvironmental targeting, and focused ultrasound and convection-enhanced delivery, concluding that meaningful progress will require biomarker-driven patient selection [5].

Nageeb and colleagues propose a compartmental framework for glioma immunotherapy, in which antitumor immunity must traverse antigen priming, trafficking and effector persistence, and therapeutic failure often reflects a mismatch between where an agent acts and where the dominant immunological barrier resides [6]. This framework renders the negative checkpoint inhibitor trials and transient chimeric antigen receptor (CAR) T-cell responses mechanistically interpretable and supports combinations sequenced by compartment. The expansion of cellular therapies is, however, accompanied by distinct neurological toxicities. Hauswirth and Dietrich review immune effector cell–associated neurotoxicity syndrome, delayed movement and neurocognitive toxicity, and tumor inflammation–associated neurotoxicity [7]. With the 2026 FDA approval of axicabtagene ciloleucel for primary CNS lymphoma, familiarity with these syndromes is now directly relevant to neurological practice.

Two contributions examine the interplay between tumor biology and patient age. There is increasing recognition that molecular drivers, rather than chronological age, determine tumor behavior: MAPK-altered low-grade gliomas, IDH-mutant gliomas and histone-mutant high-grade gliomas arise across the pediatric–adult continuum. Nevertheless, the age of the patient continues to dictate the care they receive and the therapies best suited to them. Brizini and colleagues characterize pediatric low-grade glioma shaped by BRAF, MEK and type II RAF inhibitors, in which treatment of the developing child raises questions regarding treatment duration, rebound growth, long-term toxicity and equitable access [8]. Hennawy and colleagues argue that adolescents and young adults (AYA), who account for approximately 14% of primary CNS tumor diagnoses, should be recognized as a distinct population [9]. Their management is frequently determined by whether they are treated in pediatric or adult health care systems, they remain underrepresented in pivotal trials, and their survivorship needs, including fertility, cognition, education and employment, are considerable. Together, these reviews call for care models and trials informed by tumor biology yet responsive to each patient’s developmental stage.

Brain metastases develop in up to 40% of patients with cancer. Sarfraz and colleagues review the intracranial efficacy of targeted therapies, immune checkpoint inhibitors and antibody–drug conjugates, the agent-specific risk of radionecrosis with stereotactic radiosurgery, and a decision framework stratified by intracranial response rates for determining when systemic therapy may appropriately precede local treatment [10].

Meningioma has likewise entered an era of integrated stratification. Yefet, Zadeh and Nassiri review how methylation, transcriptomic and cytogenetic classifiers converge on biological groups with distinct recurrence risk, and how quantitative MRI, somatostatin receptor PET and computational pathology complement histopathological grading [11]. Therapeutically, the phase 3 ROAM/EORTC-1308 trial demonstrated that adjuvant radiotherapy approximately halved recurrence following gross total resection of WHO grade 2 meningioma, and ongoing trials will evaluate molecularly guided radiotherapy and 177Lu-DOTATATE.

Finally, Mendonca and colleagues examine extracellular vesicles as a therapeutic platform for glioblastoma [12]. These nanovesicles can traverse the blood–brain barrier, carry diverse molecular cargo and modulate antitumor immunity, but their clinical translation is limited by the absence of standardized isolation protocols, scalable manufacturing and efficient CNS targeting.

Several themes emerge from these contributions. First, molecular diagnostics have matured from a tool for classification into a determinant of therapy: methylation profiling and refined grading underpin diagnosis [1], molecularly selected targeted agents have entered practice [4], [8], and intraoperative sequencing and cerebrospinal fluid liquid biopsy promise to extend molecular information to the operating room and the surveillance clinic [3], [8], [9]. Second, trial design is evolving in step with this biology through master protocols, adaptive designs and biomarker-selected platforms, although these will succeed only when paired with active, CNS-penetrant agents [2], [11]. Third, biomarker discovery increasingly extends beyond tissue. Amino acid PET can delineate infiltrative glioma beyond the contrast-enhancing margin to guide resection and help distinguish progression from treatment-related change during targeted therapy [3], [8], while somatostatin receptor PET in meningioma both guides surgical and radiotherapy planning and selects patients for 177Lu-DOTATATE, a theranostic paradigm in which the imaging biomarker identifies the therapeutic target [11]. Fourth, efficacy depends as much on delivery as on the agent, whether across the blood–brain barrier, into the relevant immunological compartment, or to patients remote from specialized centers [5], [6], [10], [12]. Finally, as survival improves, the neurological, cognitive and psychosocial sequelae of disease and treatment warrant the same methodological rigor applied to survival endpoints [7], [9].

Although formidable challenges remain, there are genuine grounds for optimism. Precise molecular diagnosis, biologically informed trial designs, and imaging and fluid biomarkers that guide surgery, radiotherapy and drug selection are converging to enable treatment that is both more effective and less toxic. The first targeted therapies have already altered the course of disease for selected patients with glioma, and the multimodal strategies now in development offer the prospect of extending these gains to a broader population, including patients with glioblastoma, while preserving neurological function and quality of life. We are grateful to the authors for their contributions, to the reviewers for their careful appraisal of each manuscript, and to the Editor-in-Chief and editorial staff of *Neurotherapeutics* for the opportunity to assemble this special issue. It is our hope that these reviews will serve both as a contemporary reference for clinicians and investigators and as a framework for future research in neuro-oncology.
